# An educational approach for early student self-assessment in clinical periodontology

**DOI:** 10.1186/s12909-021-03078-9

**Published:** 2022-01-12

**Authors:** Shaun Ramlogan, Vidya Raman

**Affiliations:** Periodontology, Restorative Unit, School of Dentistry, Faculty of Medical Sciences, The University of the West Indies, St Augustine Campus, Eric Williams Medical Sciences Complex, Uriah Butler Highway, Champs Fleurs, West Indies Trinidad and Tobago

**Keywords:** Periodontology, Clinical skills, Education, Cognitive, Self-assessment

## Abstract

**Background:**

Self-assessment is a mandated educational requirement for use in dental undergraduate programmes. It is weakly supported for use in early clinical training and studies are criticized for the conceptual and methodology shortfalls. The aim of the study was to compare the alignment of student self-assessment to both staff assessment and written exams in early clinical training using an educational approach.

**Methods:**

In 2014-2015, 55 third-year dental students completed three educational sessions comprising of (a) classroom teaching (lecture, video) with post-lesson written exam and (b) clinical activity with student self-assessment, staff assessment and student reflection. An intra-individual analysis approach, staff validation, and student scoring standardization were implemented. Cognitive (clinical competency) and non-cognitive (professionalism) items were separated in the analyses.

**Results:**

There were medium correlations (Spearman’s rho, r) between student self-assessment and staff assessment scores for cognitive items (r, 0.32) and for non-cognitive items (r, 0.44) for all three combined sessions. There were large correlations for individual sessions. Compared to the post-lesson written exam, students showed small correlation (r, 0.22, 0.29) and staff showed medium correlation (r, 0.31, 0.34) for cognitive and non-cognitive items. Students showed improvements in their mean scores for both cognitive (t-test; *p* > 0.05) and non-cognitive items (t-test; *p* = 0.000). Mean scores of students were not different statistically from that of staff (*p* > 0.05).

**Conclusions:**

Students may adequately act as self-assessors at the beginning of their clinical work in periodontology. Self-assessment may potentially improve the clinical performance. Self-assessment may be nurtured through clear guidelines, educational training strategies, feedback and reflection leading to better evaluative judgement and lifelong learning.

## Background

Self-assessment was broadly defined as an individual’s psychological evaluation of one’s quality of work in relation to internal factors (e.g. self-image, academic self-concepts, motivation), interpersonal factors (e.g. relationships with peers, instructors) and external and perceived standards (e.g. culture, norms) [1]. The self-assessment process involved an individual’s collection, evaluation and reflection on the quality of learning, performance and outcome against internal (e.g. values, ideas, goals, skills, emotions) and external (e.g. teachers, tests) criteria with feedback and determination of one’s strengths and weaknesses [2, 3]. Self-assessment was a valuable quality assurance tool in education as the learner directly evaluated his or her attainment of the learning objectives. The General Dental Council’s ‘Standards for Education’ document included self-assessment as an important example of clinical competency evidence during undergraduate education in the United Kingdom (UK) [4]. Additionally in the United States of America (USA), the Commission on Dental Accreditation (CODA) of the American Dental Association (ADA) included students’ self-assessment as an important accreditation standard of dental education programmes [5]. Development of self-assessment skills has been a purported competency goal of every education programme where competency was defined as the ability to self-assess [6]. One review on the evaluation of clinical competency in dentistry proposed continuous low-stake formative assessments over single high-stake summative assessments to improve student engagement and learning [7].

Well-structured self-assessment programmes have been reported to create ‘productive learning environments’ lending to ‘more mature and collegial’ health professionals [8]. In a review study of self-assessment use in 11 health profession training programmes, there were reported cognitive benefits of ‘improvements in knowledge, performance, and self-analysis of performance’. [8] There were also non-cognitive benefits such as ‘improvements in morale, motivation, and communication’. Cognitive skills involved conscious intellectual effort exemplified by synthesis of memory, information processing, knowledge, intelligence, mathematical reasoning, motor skills and problem solving. Non-cognitive skills involved less conscious effort exemplified by motivation, integrity, responsibility, social skills, personality and the attitude [9].

A small yet significant association was reported in a study comparing non-cognitive traits in UK Clinical Aptitude Test (UKCAT) to subsequent clinical performance of medical students between 2007 and 2010 [10]. Self-assessment studies which rely solely on cognitive aspects may have limitations for validity as students and staff view cognitive and non-cognitive aspects differently [11, 12]. Students tended to concentrate more on non-cognitive skills while conversely staff tended to concentrate more on cognitive skills [11, 12]. Thus it was useful to separate cognitive items from non-cognitive items when investigating self-assessment.

Self-assessment was more valuable for practical skill in clinical training compared to knowledge based activity as the former was more objective and more difficult to query [13]. Additionally, higher correlations of scorings of students versus staff were associated with more specific clinical tasks [14]. Self-assessment scorings were better suited for short, structured, simple tasks compared to longer tasks. It was also more appropriate for shorter time intervals such as a given clinical session compared to longer time intervals such as clinical rotations [2, 12]. Tai, Ajjawi, Boud et al. (2018) proposed that self-assessment as a pedagogical strategy was underpinned by evaluative judgement which was broadly defined as the ‘capability to make decisions about the quality of work of self’. [15]. Evaluative judgement as first applied to clinical medical students involved critical assessment of performance against an explicit standard and complex reflection [16]. While self-assessment was task related, evaluative judgement related to life-long learning and higher cognitive goals [15].

Quantitative self-assessment in general research has been cited to have conceptual and methodology limitations with recommendations for more explicit study descriptions of both independent variables (e.g. population characteristic, study characteristics, rating criteria, scale reliability) and dependent variables (e.g. degree of agreement and distribution) [17]. Student self-assessment in medical research has been reviewed in the literature as underdeveloped, weakly supported and also limited in practical application due to methodology shortfalls [6, 8, 12–14, 18]. The first methodology shortfall was the comparison of the self-assessor to the staff or expert gold standard. This gold standard of staff assessment has been cited as an unreliable valid measure of the assessed performance dimension. The difficulty related to the staff having a good understanding of the assessment dimension (knowledge or skill) relative to the actual performance of the student. One reviewer group has suggested optimizing of the reliability of the expert or staff scoring by use of multiple scorers and applying a ‘correction for attenuation’ formula for correlation as proposed by Regehr et al. [2, 12]

The second methodology shortfall was the different interpretation and use of the scoring scale by each student [8, 12, 14]. Boud and Falchikov (1989) cited problems with the use of different rating scales, unclearly defined rating criteria and undefined expectations with students ratings as the students scored either effort rather than performance or generated impressionistic scores of what the teacher would have given them [17]. This error was reduced by having clearly defined responses and guidance for the scoring scale. Accuracy and validity of self-assessment was improved by having graded or explicit self-assessment criteria, benchmarking guidance and training strategies [8, 13, 14, 18, 19]. Feedback and discussion have engendered improved knowledge of the task and awareness of the ideal standard or performance [13].

The third methodology shortfall was group level analyses with student comparison to one another despite individual differences in both application and competency of self-assessment [12, 13]. An intra-individual approach with student evaluation over multiple tasks as a measure of individualized self-assessment accuracy ameliorated this shortfall [12].

In a survey on undergraduate periodontal education and assessment of 76 European dental institutes within member countries of the European Federation of Periodontology (EFP), the authors reported that self-assessment was rarely used [20]. There have been few reports of the use of self-assessment for practical clinical periodontology within the undergraduate dental programme [21–23]. Two of these studies compared student to staff and emphasized the need for feedback for better self-assessment [22, 23]. The third study utilized both written exam and faculty comparisons and reported some correlation for treatment skill [21]. Accurate self-assessment required comparison to some validated or appropriate criteria [14].

Student self-assessment was weakly supported by the literature and many studies were criticized for conceptual and methodology limitations. Despite this there was a mandate for its use in educational programmes in dentistry and frequently it was implemented in clinical training. It was reported that self-assessment skills improved with time [24]. However the value of the use of self-assessment at the start of clinical training was undetermined. Further, there was a need to close the gap between the alignment of student and staff evaluation for clinical activities based on the recommendations from the reviewed literature.

Thus the aim of this study was to investigate the use of student self-assessment in early clinical training in periodontology. The main objectives were to compare (a) student self-assessment scores to staff assessment scores and (b) student self-assessment scores or staff assessment scores to student performance in written exams. Further objectives to address reported methodology shortfalls were to utilize educational training strategies for student scoring and benchmarking, validation of the expert/staff scoring, determination of scale reliability and an intra-individual analysis approach.

## Methods

Fifty-five dental students during the third-year of their undergraduate degree programme (DDS; Doctor of Dental Surgery) were all included from two consecutive graduating classes of 2016 (*n* = 27) and 2017 (*n* = 28) during the period 2014-2015. These students were at their respective times registered for an introductory clinical periodontology course at the start of clinics at the School of Dentistry, Faculty of Medical Sciences, St Augustine Campus, The University of the West Indies (UWI), Trinidad and Tobago. A pre-requisite second-year course, covering basic periodontal knowledge and skills in the phantom-head laboratory, had been completed. This study was exempted from ethical approval by the UWI Campus Ethics Committee.

The introductory clinical periodontology course comprised of three educational sessions with the following topics: (1) Full Mouth Plaque Score (FMPS) covering plaque assessment, (2) Basic Periodontal Examination (BPE) covering periodontal disease screening and (3) Full Periodontal Examination (FPE) covering detailed periodontal disease assessment. All three educational sessions occurred over a one-month period. Each educational session involved both classroom and clinical activities.

**Fig. 1:**
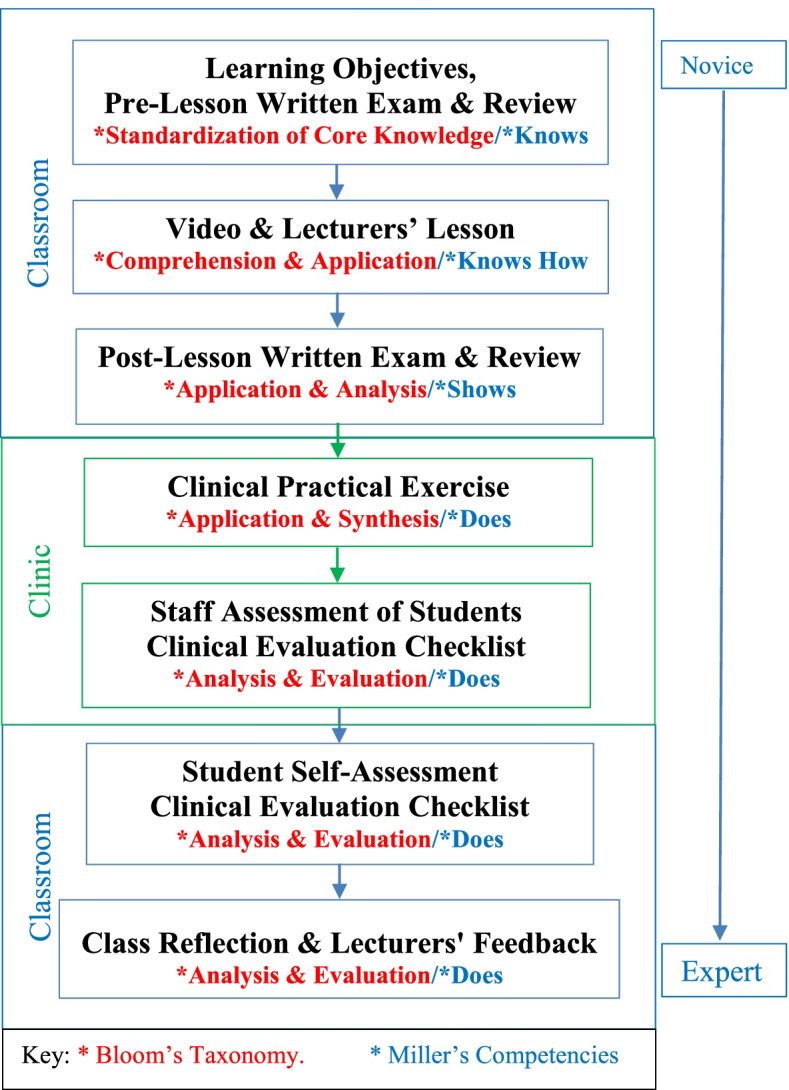
Flow diagram of methodology per educational session

Self-assessment was reported as an important component of critical thinking at a higher cognitive order to improve self-directed learning [24]. The pedagogical teaching/learning strategy in the methodology was to progress along the cognitive domains of Bloom’s taxonomy (knowledge, comprehension, application, analysis, synthesis and evaluation) to ensure that students had acquired the guidance for self-assessment [25]. Bloom’s taxonomy was exemplified in this study by acquisition of knowledge (pre-lesson written exam and review), comprehension (video and lecture), application (video practical, post-lesson written exam, practical exercise), analysis (exam review, self-assessment, staff assessment), synthesis (clinical practical application) and evaluation (self-assessment, staff assessment, reflection and feedback) (Fig. 1). Application of Miller’s pyramid of clinical competence (knows, knows how, shows and does from novice to expert respectively) to the practical activities was also integrated as an educational strategy [26]. Miller’s clinical competencies were also represented in parallel to Bloom’s by knows (pre-lesson written exam and review), knows how (video and lecture), shows (post-lesson written exam and review) and does (practical, self-assessment, staff assessment and feedback). This methodology per educational session is illustrated with the flow diagram in Fig. 1.

The student self-assessment was a formative assessment and served to facilitate continuous feedback for students to identify strengths and weaknesses and enable self-regulated learning. The student self-assessment did not contribute to the final grade of the course. This grade was derived from a summative written exam.

The staff members were two lecturers in periodontology who were both present for all components of the sessions. Each classroom component began with the distribution of the learning objectives, a pre-lesson short-answer written exam and then review to consolidate second-year learning of fundamental core terms and concepts.

The lesson comprised of viewing of a clinical instructional video, lecture and class discussion. The 12-14 min long videos were initially created for the class of 2015 and the video methodology details have been previously reported [27]. Learning objectives covered by the video included knowledge, comprehension and application of instruments, clinical charts and methodology via phantom-head jaw or live patient demonstration. Videos were viewed once per session and not posted on the Moodle-based ‘Myelearning’ or other internet platforms. This was to standardize the educational exposure and prevent future years from viewing the video ahead of time. The video instruction was reinforced by lecturers’ discussion with the class. Thus all sessions utilized a combination of video and lecturers’ instruction.

A post-lesson written exam followed to determine the learning achieved by the students. Exam items covered instruments, methodology and clinical charting inclusive of tooth and site recognition. This written exam was designed to test competencies related to clinical practice and was mapped to the lesson learning objectives. An application example of this mapping for the learning objective: ‘students should be able to correctly use the BPE probe for periodontal screening’ was related to the exam items of (a) diagrammatic representation of correct orientation of the BPE probe and (b) reading and interpreting the BPE probe. The written exams were piloted over the previous two years. The correlation strength between the past written exams was medium (Spearman’s rho, r = 0.46; *p* = 0.015) to large (r = 0.52; *p* = 0.004) thus endorsing these exams. Post-lesson written exam scripts were submitted for staff grading according to a master answer sheet. The post-lesson exam was immediately reviewed with the class for feedback and discussion for further knowledge standardization. Prior to the clinical exercise, the objectives and instructions for the clinical component were shared via printed material and discussed within the classroom.

In the practical exercise of the clinical component, students were assigned to one dental operatory in groups of 3 to 4 and each student acted in turn as an operator or consented patient without staff assistance or intervention. Staff assessment of each student operator was achieved by completion of a clinical evaluation checklist (see Table 1). Direct feedback to students from the staff at the clinical exercise was avoided so as not to influence or bias the ensuing student self-assessment. The smaller class size allowed for closer student monitoring and identification of the weaker students through direct observation by staff in the clinic. Direct feedback to students from the staff was reserved for the final reflective session.

**Table 1:** Clinical evaluation checklist

Lesson 1: Full Mouth Plaque Score (FMPS)				
**FMPS Procedure**	**Rank (Please circle one)**			
**I was able to:**				
select the mirror and the explorer	3 Excellent	2 Good	1 Weak	0 Fail
visualize plaque on my explorer	3 Excellent	2 Good	1 Weak	0 Fail
identify the four surfaces per tooth	3 Excellent	2 Good	1 Weak	0 Fail
complete the clinical plaque scoring	3 Excellent	2 Good	1 Weak	0 Fail
complete the plaque charting	3 Excellent	2 Good	1 Weak	0 Fail
calculate the FMPS using the formula (without decimal places)	3 Excellent	2 Good	1 Weak	0 Fail
**Professional Practice**				
**I was able to:**				
practice proper professional dress and attitude	3 Excellent	2 Good	1 Weak	0 Fail
practice proper infection control and safety	3 Excellent	2 Good	1 Weak	0 Fail
practice proper time management	3 Excellent	2 Good	1 Weak	0 Fail
practice proper team work	3 Excellent	2 Good	1 Weak	0 Fail

This clinical evaluation checklist was developed by both staff members and piloted in the previous year. Staff members were to be guided and calibrated by the same instructions as for the students to standardize the use of the checklist as detailed later in the methodology. The inter-rater reliability was calculated for the two staff members for a group of common students (*n* = 15) and showed good Kappa measure of agreement of 0.70.

After the clinical exercise, within the classroom each student independently completed his/her clinical evaluation checklist to self-assess his/her own performance. Class discussion ensued where they identified their clinical strengths and weaknesses. The intention was to maintain the student independence of the self-assessment process by having a student driven discussion with students also learning from each other. Individual student participation in feedback and discussion was also facilitated by the smaller class. This critical evaluation was then supplemented with direct feedback from both staff members with the intension of improving clinical guidance. The staff feedback was the final quality control which aimed to identify and guide weaker student performances. Staff feedback which was related to the learning objectives also guided discussion on clinical performance issues missed by the students.

Students were familiar with Likert scale questionnaires as they had used these in the past for course evaluations. The four-point Likert scale clinical evaluation checklist comprised of ten questions. The central neutral response was omitted to provide a ‘forced choice’ with balanced keying options. The first six cognitive items were clinical competency-based questions on the ability to complete the practical task and chart recording while the next four non-cognitive items were professionalism-based items included attire, infection control, time management and team work. Neither students nor staff were clued into further definition of these non-cognitive items to limit any bias in scoring personal attributes. However, the inherent non-cognitive characteristics may be defined but not limited to as follows: attire – discipline, non-verbal communication of professionalism; infection control – self-discipline of reduced risky behaviour; time management – attention, planning, persistence; team work – cooperation, interpersonal skill and verbal communication.

The numerical scores and question details of the clinical evaluation checklist were clearly explained and described to the students as follows: 3 excellent- student has mastered item and needs little or no correction, 2 good – student has performed adequately but requires some correction, 1 weak- student requires significant correction and 0 fail – student’s performance is completely unsatisfactory or unable to complete the task.

The clinical expectations of the upper limit scoring of 3 was standardized via the video and lecturers’ lesson. It was felt that further attempts to standardize the lower scores for clinical activity would interfere with the self-assessment process of the students as well as negate attempts to validate the self-assessment for the given clinical scenario.

Over all three educational sessions, each student had 18 cognitive scores (clinical competence) and 12 non-cognitive scores (professionalism) as determined separately by the student and staff. Thus allowing for intra-individual analyses for each student along all self-assessments exercises compared to staff. The cognitive and non-cognitive Likert scale questions (clinical checklist) were tested for internal consistency (reliability) via Cronbach’s alpha. There was a very high reliability for the cognitive score (0.857) and high reliability for the non-cognitive scale (0.789).

SPSS version 24 statistical software (IBM Corporation, Armonk, New York, USA) was used for the data analyses. Mean scores per session for student and staff were calculated and the statistical difference determined by Analysis of Variance (ANOVA) (*p* < 0.05) for cognitive (clinical competence) and non-cognitive items (professionalism) separately.

Emphasizing the intra-individual approach, statistical correlation comparisons determining Spearman’s rho correlation coefficients (*p* < 0.05) were also obtained for pairs of scores between each individual student and staff for separate categories of cognitive (clinical competency) and non-cognitive (professionalism) items for each of the three sessions. The student-staff Spearman’s rho correlations coefficients (r) were optimized for reliability by ‘correction for attenuation’ formula by dividing the coefficients by the square root of the staffs’ interrater reliability [2].

The relationships between post-lesson exam grade and (a) student self-assessment score and (b) staff assessment were also explored via Spearman’s rho correlation coefficient at a significance of *p* < 0.05 for all educational session for categories of a) cognitive (clinical competence) and (b) non-cognitive (professionalism).

The strength of relationship for Spearman’s rho correlation coefficient (r) was categorized as follows: small correlation as r = 0.10 to 0.29; medium correlation as r = 0.30 to 0.49 and large correlation as r = 0.50 to 1.0 [28].

## Results

A total of 55 students consented and were included in the study. The students comprised of 17 (31%) males and 38 (69%) females with a mean age of 22.5 years (range 20-33 years). The percentage attendance at each session was high at 96.4% (53 students) for FMPS, 94.5% (52 students) for BPE and 90.9% (50 students) for the FPE session.

**Fig. 2:**
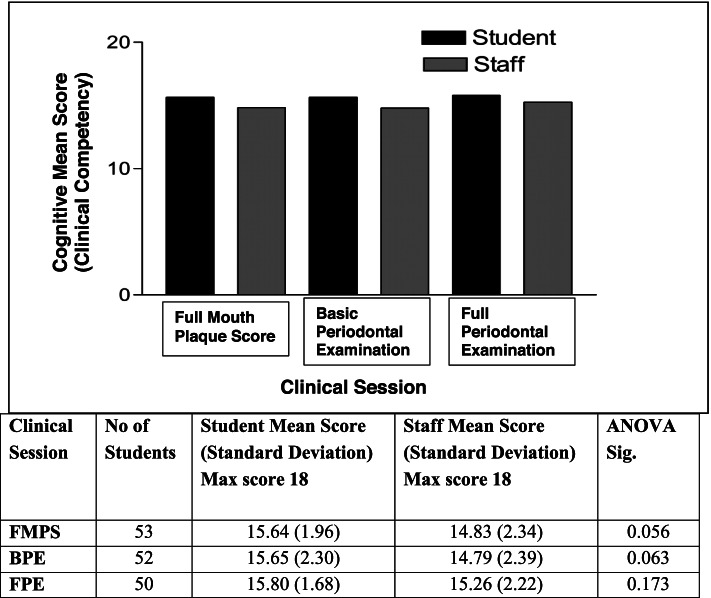
Student and staff mean scores for cognitive items (clinical competency) per session

Figure 2 shows the mean scores out of 18 for cognitive items (clinical competency) by student self-assessment and by staff assessment for the three clinical sessions of FMPS, BPE and FPE as determined through the clinical evaluation checklist. The mean scores for all three clinical sessions were higher as determined by students compared to that determined by staff. However, there was no statistically significant difference between these scores by students and staff for each clinical session (ANOVA; each session *p* > 0.05).

**Fig. 3:**
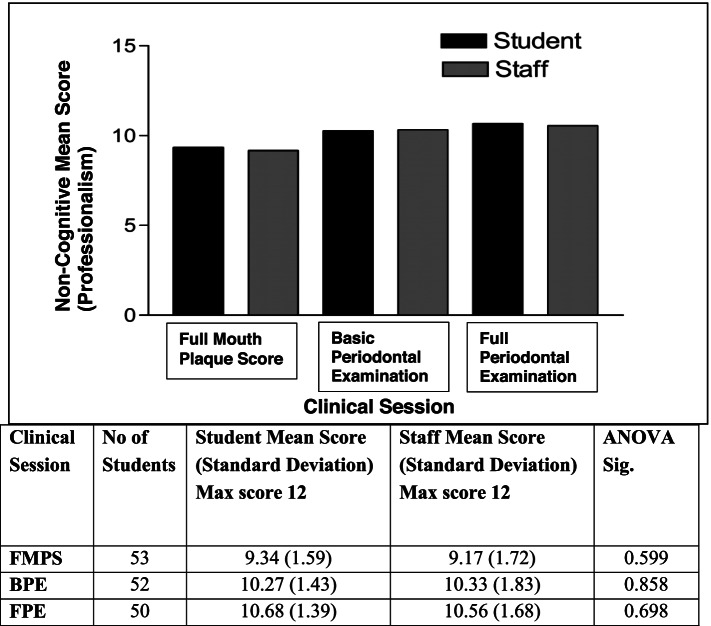
Student and staff mean scores for non-cognitive items (professionalism) per session

Figure 3 shows the mean scores out of 12 for non-cognitive items (professionalism) by student self-assessment and by staff assessment for the three clinical sessions of FMPS, BPE and FPE as determined through the clinical evaluation checklist. The mean scores as determined by students were higher for FMPS and FPE but lower for the BPE session compared to that determined by staff. There was also no statistically significant difference between these mean scores by students and staff for each of clinical sessions (ANOVA; each session *p* > 0.05).

The means scores for non-cognitive items (professionalism) from the first session (FMPS) to the last session (FPE) showed an increase from 9.34 to 10.68 as assigned by students and 9.17 to 10.56 as assigned by staff. Both these increases were statistically significant in the comparison of the means (t-test; *p* = 0.000). While there were also small improvements in cognitive items (clinical competency) from the first to the last session, this improvement was not statistically significant.

The relationship between the individual student scores (intra-individual approach) for cognitive items (clinical competency) and non-cognitive items (professionalism) as determined by student and as determined by staff was also explored via Spearman’s rho correlation coefficient (r) at a significance level of *p* < 0.05. The corrected Spearman’s rho correlation coefficients were determined by dividing by the square root of the staffs’ interrater reliability of 0.70 (Kappa measure of agreement). The raw and corrected mean Spearman’s rho correlation coefficients (r) are shown in Table 2 for cognitive items (clinical competency) and Table 3 for non-cognitive items (professionalism). Statistical analyses were only possible where there were valid pairs of data with differences in the scores per student or per staff.

**Table 2:** Spearman Correlation between student self- and staff assessment for cognitive items

Session	Cognitive Items			
	Raw Spearman’s Correlation		Corrected Spearman’s Correlation	
	Mean Coefficient (Number of valid pairs)	Standard Error of Mean (95% Confidence Interval)	Mean Coefficient (Number of valid pairs)	Standard Error of Mean (95% Confidence Interval)
FMPS	**0.39** (24)	0.05 (0.28-0.50)	**0.46** (24)	0.06 (0.33-0.60)
BPE	**0.49** (27)	0.06 (038-0.61)	**0.59** (27)	0.07 (0.45-0.73)
FPE	**0.42** (28)	0.05 (0.32-0.53)	**0.51** (28)	0.06 (0.38-0.64)
All 3 Sessions	**0.27** (45)	0.02 (0.22-0.31)	**0.32** (45)	0.03 (0.27-0.38)

**Table 3:** Spearman Correlation between student self- and staff assessment for non-cognitive items

Session	Non-Cognitive Items			
	Raw Spearman’s Correlation		Corrected Spearman’s Correlation	
	Mean Coefficient (Number of valid pairs)	Standard Error of Mean (95% Confidence Interval)	Mean Coefficient (Number of valid pairs)	Standard Error of Mean (95% Confidence Interval)
FMPS	**0.57** (38)	0.04 (0.48-0.66)	**0.68** (38)	0.05 (0.58-0.78)
BPE	**0.51** (17)	0.07 (035-0.67)	**0.60** (17)	0.08 (0.42-0.78)
FPE	**0.64** (9)	0.07 (0.48-0.81)	**0.77** (09)	0.09 (0.57-0.97)
All 3 Sessions	**0.37** (50)	0.03 (0.31-0.42)	**0.44** (50)	0.03 (0.38-0.51)

For cognitive items (clinical competency), the raw mean r was 0.27 (small correlation) and corrected mean r was 0.32 (medium correlation) for all three sessions (*n* = 45). While individual sessions presented with medium correlations (0.39, 0.49, 0.42) for raw mean r and medium to large correlations (0.46, 0.59, 0.51) for corrected mean r (Table 2).

For non-cognitive items (professionalism), the raw mean r was 0.37 (medium correlation) and corrected mean r was 0.44 (medium correlation) for all three sessions (*n* = 50). However individual sessions presented with large correlations for both raw (0.57,0.51, 0.64) and corrected (0.68, 0.60, 0.77) mean r (Table 3).

The relationship between the students’ post-lesson exam grades and the mean scores for cognitive items (clinical competency) as determined by student and as determined by staff were also explored via Spearman’s rho correlation coefficient (r) at a significance level of *p* < 0.05. For cognitive items (clinical competency), the mean r was 0.22 (small correlation) for student and post-lesson exam grades comparison and 0.31 (medium correlation) for staff and post-lesson exam grades comparison for all three sessions. For non-cognitive items (professionalism), the mean r was 0.29 (small correlation) for student and post-lesson exam grades comparison and 0.34 (medium correlation) for staff and post-lesson exam grades comparison for all three sessions. There was a general trend for improvement in the correlation values between student self-assessment and staff assessment with time as viewed from the first session (FMPS) to the last session (FPE) for both cognitive and non-cognitive items.

## Discussion

Overall, there were medium to large correlations between student and staff scores for explicitly defined clinical activity in early clinical training in this study. There were improvements in mean scores derived by both student and staff as the sessions progressed possibly due to the self-assessment exercise combined with the inherent educational training, guidance and feedback. In this formative educational activity, students’ ability to self-assess improved with time as there was a general trend for higher correlation values between student and staff over the three sessions. Metz et al. (2017) reported this improvement with time and Boud and Falchikov (1989) suggested that self-assessment may be more suited for formative type rather than summative type evaluations as the latter produce inflated scoring [17, 24]. This study’s findings suggested that within its methodology protocols with an educational approach there was a satisfactory application for self-assessment for early clinical training.

For group analyses, students were able to assign scores which were similar (no significant difference) to the staff expert standard. For intra-individual analyses with staff scoring validation, there were overall medium correlations between student and staff scores for all three session and medium to large correlations for individual sessions. This further supported that students were adequate self-assessors when comparing the student’s ability to the corrected staff standard.

The higher correlations for non-cognitive (professionalism) compared to cognitive items (clinical competency) related to the literature where students were noted to emphasize non-cognitive items compared to cognitive items [11, 12]. The higher correlations in the individual sessions compared to all three sessions may be due to the lower numbers of valid correlation pairs due to statistical exclusion. The drawback of this statistical methodology was the exclusion of pairs in which there was constant grading for all items.

For comparison to an external standard of the written exam, student self-assessment showed small correlation and staff assessment showed medium correlation. However, these correlation coefficient values were very close to each other and numerically the student value just fell outside the medium correlation range. The difficulty in interpretation of this finding may relate to the merit in using this written exam as an appropriate measure. While this written exam was endorsed through prior use and determination of correlation strength (Spearman’s rho), it was limited by the fact that the knowledge of the students had now changed following review of the written exam. Thus the written exam was an inherent part of the process for improved self-assessment through improved feedback and guidance.

Students showed improvements in clinical practice through improved mean score of the clinical checklist from one session to the next. This gain was only statistically significant for non-cognitive (professionalism) items. Colthart et al. (2008) noted that accuracy of self-assessment improved with feedback and guidance but also reported no studies in their review looked at changes in clinical practice through self-assessment [13]. The short duration of one month in our study may have precluded any statistically significant changes in cognitive items (clinical competency).

The strengths of the study included: (1) clearly defined and specific short clinical task (2) explicit clinical evaluation checklist and reliable scale (3) educational strategies for training, guiding and benchmarking student scoring (4) separation of cognitive (clinical competency) and non-cognitive (professionalism) items (5) expert/staff validation via multiple staffs’ interrater reliability and ‘correction for attenuation’ formula (7) comparison of self-assessment to both staff assessment and written exam (inclusion of both mean and correlation values) (8) determination of improvement in clinical scores. The explicit criteria, the benchmarking and determination of scale reliability supported the replication of the study for consecutive year III clinical years.

Several authors have expressed issues with the lack of understanding of the ‘nature and role’ of self-assessment with the need to concentrate on the ‘individual’s cognition in developing clinical competence’. [13, 29] However the pedagogical strategies with feedback and self-reflection based on the student self-assessment in this study not only fostered better self-assessment but also a tendency to better clinical activity.

The weaknesses of this study included: (1) no sampling strategy and single institution investigation (2) small sample size (3) no consideration of gender, culture and level of performance.

The study was limited by the usual educational and clinical activities within the dental curriculum at the School of Dentistry, UWI. Thus there wasn’t consideration or opportunity for sampling or inclusion of other institutions. The small sample size was related to the usual class enrolment and facilitated direct clinical monitoring and feedback. There was no attempt to determine the effect of potential variables such as gender, culture and level of performance as these were variables that could not be altered or matched in any given class. Generally, students in dental school have already undergone a rigorous selection criteria for entry and are thus are of higher academic capabilities. The intension of the study was to explore the application and alignment of early student self-assessment and staff assessment in a clinical environment considering many of the reported conceptual and methodological issues in the literature.

These third-year dental students were at the beginning of their clinical training with their first patient encounter in this study. As part of their future clinical activities, they are expected to grade (self-assess) themselves for each session prior to being graded by staff. Thus it was important to not only expose and initiate training in the self-assessment process but also to determine the useful application of this method in early clinical training. Self-assessment application to educational programmes with expanding enrolments may act as a further mechanism for quality control and expediency.

Students’ ability to self–assess relates to their ability to judge their own work and make clinical decisions based on the delivered standard of care. The advantage of such a strategy is the aiding of the production of practitioners who are able to think and operate independently and fosters evaluative judgement.

With graduation, these dental students transition from a supervised and supported position during their undergraduate training to an unsupervised and occasionally isolated practice post-graduation. Thus to facilitate this weaning from institutional support, it is important that students develop their self-assessment skills.

Confusions in the understanding of the ‘nature and role’ of self-assessment necessitate defining this role and purpose [13, 29]. In our study if the role of self-assessment was to discern good students from weak students then the self-assessment task adequately fits the purpose. However, if the role was to give a higher level of discernment related to detailed performance example: marks out of 100, then the self-assessment task definitely does not fit the purpose. Ideally, self-assessment in this study was used to determine competent students who have adequate skills and help students reflect on their performances for improvement.

Self-assessment was an integral part of student educational development in their clinical training. It should not be thought of as a replacement for staff support, guidance and evaluation. It also should not be viewed as the only strategy for clinical improvement but should be valued as part of the requirement for student reflection and professional growth.

Boud and Falchikov (1989) also suggested that efforts to maximize agreement between student and staff assessment should be directed to systematic formative course activities which sensitize students to their own work [17]. This UWI study adopted these recommendation through design and application to the early clinical scenario for improvement of accuracy and quality of self-assessment. Continued self-assessment facilitates better evaluative judgement as a cumulative and life-long goal of this educational exercise [15].

Follow-up work in this study may evaluate the students’ ability to self-assess at both the beginning and the end of their clinical training. This may be linked to longer term changes in clinical practice competency and attitudes to both self-assessment and clinical practice. Studies with larger cohorts may facilitate exploration of student characteristics such as age, gender and ability which may impact on self-assessment capabilities. Additionally comparisons of self-assessment to peer assessment in a clinical setting may be made and how it relates to group learning or education.

## Conclusion

Students were deemed to act as adequate self-assessors at the beginning of their clinical work in periodontology when compared to both staff and written examination. The process of self-assessment and evaluative judgement for short clinical tasks may be nurtured through clear explicit guidelines which hinge on educational training strategies involving classroom instruction, written examination, discussion, feedback and reflection. Exposure and practice though this strategy helped to foster better self-assessment as well as clinical performance. Educators should be motivated to use and develop accurate and quality self-assessment for clinical activity through an educational formative approach, feedback and reflection.

## Data Availability

The datasets generated and/or analysed during the current study are not publicly available due student confidentiality but are available from the corresponding author on reasonable request.

## Abbreviations

ADA: American Dental Association
ANOVA: Analysis of Variance
BPE: Basic Periodontal Examination
CODA: Commission on Dental Accreditation
DDS: Doctor of Dental Surgery
EFP: European Federation of Periodontology
FMPS: Full Mouth Plaque Score
FPE: Full Periodontal Examination
IBM: International Business Machines
r: Spearman’s rho
SPSS: Statistical Product and Service Solutions
USA: United States of America
UK: United Kingdom
UKCAT: UK Clinical Aptitude Test
UWI: The University of the West Indies

## References

[1] Andrade HL and Brown GTL. 11 Jul 2016, Student self-assessment in the classroom from: handbook of human and social conditions in assessment Routledge. At: https://www.routledgehandbooks.com/doi/10.4324/9781315749136.ch18. Accessed on: 01 Sep 2021.

[2] Regehr G, Hodges B, Tiberius R, Lofchy J (1996). Measuring self-assessment skills: an innovative relative ranking model. Acad Med.

[3] Yan Z, Brown G (2016). A cyclical self-assessment process: towards a model of how students engage in self-assessment. Assess Eval High Educ.

[4] General dental council; standards for education: standards and requirements for providers. 2015. At: www.gdc-uk.org/docs/default-source/quality-assurance/standards-for-education-(revised-2015).pdf?sfvrsn=1f1a3f8a_2. Accessed: 1st May 2020.

[5] Commission on Dental Accreditation. Self-study guide for dental education programs. Standard 2; page 36. American Dental Association, Chicago, Illinois. 2012. At: www.ada.org/~/media/CODA/Files/predoc.ashx. Accessed: 1^st^ May 2020.

[6] Gadbury-Amyot CC, McCracken MS, Woldt JL, Brennan RL (2014). Validity and reliability of portfolio assessment of student competence in two dental school populations: a four-year study. J Dent Educ.

[7] La Chimea T, Kanji Z, Schmitz S (2020). Assessment of clinical competence in competency based education. Can J Dent Hyg.

[8] Gordon MJ (1992). Self-assessment programs and their implications for health professions training. Acad Med.

[9] Ren T, Yu X, Yang W. Do cognitive and non-cognitive abilities mediate the relationship between air pollution exposure and mental health? PLoS One. 2019;14(10):e0223353. 10.1371/journal.pone.0223353 PMC6808496 31644533

[10] Finn GM, Mwandigha L, Paton LW, Tiffin PA (2018). The ability of 'non-cognitive' traits to predict undergraduate performance in medical schools: a national linkage study. BMC Med Educ.

[11] Arnold L, Willoughby TL, Calkins EV (1985). Self-evaluation in undergraduate medical education: a longitudinal perspective. J Med Educ.

[12] Ward M, Gruppen L, Regehr G (2002). Measuring self-assessment: current state of the art. Adv Health Sci Veduc Theory Pract.

[13] Colthart I (2008). Bagnall, Evans a et al. the effectiveness of self-assessment on the identification of learner needs, learner activity, and impact on clinical practice; BEME guide no. 10. Med Teach.

[14] Gordon MJ (1991). A review of the validity and accuracy of self-assessments in health professions training. Acad Med.

[15] Tai J, Ajjawi R, Boud D, Dawson P, Panadero E (2018). Developing evaluative judgement: enabling students to make decisions about the quality of work. High Educ.

[16] Tai JHM, Canny BJ, Haines TP (2016). The role of peer-assisted learning in building evaluative judgement: opportunities in clinical medical education. Adv in Health Sci Educ.

[17] Boud D, Falchikov N (1989). Quantitative studies of student self-assessment in higher education: a critical analysis of findings. High Educ.

[18] Mays KA, Branch-Mays GL (2016). A systematic review of the use of self-assessment in preclinical and clinical dental education. J Dent Educ.

[19] Sattheesh KM, Brockmann LB, Liu Y, Gadbury-Amyot CC (2015). Use of an analytical grading rubric for self-assessment: a pilot study for a periodontal Oral competency examination in Predoctoral dental education. J Dent Educ.

[20] Gürsoy M, Wilensky A, Claffey N, Herrera D, Preshaw PM, Sanz M, Schlagenhauf U, Trombelli L, Demirel K (2018). Periodontal education and assessment in the undergraduate dental curriculum-a questionnaire-based survey in European countries. Eur J Dent Educ.

[21] Mattheos N, Nattestad A, Falk-Nilsson E, Attström R (2004). The interactive examination: assessing students' self-assessment ability. Med Educ.

[22] Oh SL, Liberman L, Mishler O. Faculty calibration and students' self-assessments using an instructional rubric in preparation for a practical examination. Eur J Dent Educ. 2018;22(3):e400-7. 10.1111/eje.12318 29266593

[23] Deeb JG, Carrico CK, Laskin DM, Koertge TE (2019). Influence of self-assessment on dental students' performance on technical assessments in periodontics. J Dent Educ.

[24] Metz MJ, Durski MT, O'Malley DeGaris M (2017). Student self-assessment of operative dentistry experiences: a time-dependent exercise in self-directed learning. J Dent Educ.

[25] Bloom BS, Engelhart MD, Furst EJ (1956). Taxonomy of educational objectives: the classification of educational goals. Handbook I: cognitive domain.

[26] Miller GE (1990). The assessment of clinical skills/competence/ performance. Acad Med.

[27] Ramlogan S, Raman V, Sweet J (2014). A comparison of two forms of teaching instruction: video vs. live lecture for education in clinical periodontology. Eur J Dent Educ.

[28] Cohen JW (1988). Statistical power analysis for the behavioural sciences (2^nd^ ed).

[29] Eva KW, Regehr G (2005). Self-assessment in the health professions: a reformulation and research agenda. Acad Med.

